# Perceived barriers and facilitators of the implementation of a combined lifestyle intervention with a financial incentive for chronically ill patients

**DOI:** 10.1186/s12875-019-1025-5

**Published:** 2019-10-18

**Authors:** C. C. M. Molema, G. C. W. Wendel-Vos, S. ter Schegget, A. J. Schuit, L. A. M. van de Goor

**Affiliations:** 1 Department of Tranzo, Scientific Center for Care and Welfare, Tilburg University, Tilburg, the Netherlands; 2 Centre for Nutrition, Prevention and Health Services, National Institute for Public Health and the Environment, Bilthoven, the Netherlands; 3 Department of Health Science, VU University, Amsterdam, The Netherlands; 4 Tilburg School of Social and Behavioral Sciences, Tilburg University, Tilburg, The Netherlands

**Keywords:** Lifestyle intervention, Physical activity, Implementation, Primary care, Chronic illness, Qualitative research

## Abstract

**Background:**

This study aims to describe barriers and facilitators of the implementation of a combined lifestyle intervention (CLI) in primary care for patients with chronic disease. The aim of CLI to help patients to create a healthy lifestyle and to maintain this healthy lifestyle. During a CLI a patient receives advice and counselling to improve health-related behavior such as physical activity and diet. Special attention was given to the influence of adding a health promoting financial incentive (HPFI) for the participants to the CLI.

**Methods:**

Twenty-four semi-structured interviews within six care groups were performed between July and October 2017. The interviews were transcribed verbatim and coded by two researchers independently.

**Results:**

Respondents mentioned several preferred characteristics of the CLI such as easy accessibility of the intervention site and the presence of health care professionals during exercise sessions. Moreover, factors that could influence implementation (such as attitude of the health care professionals) and preconditions for a successful implementation of a CLI (such as structural funding and good infrastructure) were identified. Overall, positive HPFIs (e.g. a reward) were preferred over negative HPFIs (e.g. a fine). According to the respondents, HPFIs could positively influence the degree of participation, and break down barriers for participating in and finishing the CLI.

**Conclusions:**

Multiple barriers and facilitators for successful implementation of a CLI were identified. For successful implementing CLIs, a positive attitude of all stakeholders is essential and specific preconditions should be fulfilled. With regard to adding a HPFI, more research is needed to identify the attitude of specific target groups towards an HPFI.

## Introduction

Increased obesity rates and decreased physical activity levels are strongly linked with increased prevalence and incidence of diabetes mellitus type 2 (DM2) [1, 2].

In the Netherlands, a so called ‘care group’ (a legal entity that is part of the primary care sector) has the responsibility to arrange and to contract all care for DM2 and cardiovascular disease (CVD) patients as prescribed through the Dutch Health Care Standards [3]. There are over 100 care groups in the Netherlands, which all operate in a specific region. A care group receives a fixed amount of money per patient from the health care insurer and has to contract all health care providers, such as general practitioners, needed to deliver the necessary care [3]. This fixed amount per patient is supposed to constitute an incentive for the care group to invest in prevention. By investing in prevention, the health status of patients might improve which can contribute to less intensive care a patient needs and fewer consults for example. Implementing combined lifestyle interventions (CLI) are such a form of prevention. A CLI is an intervention that aims to help patients change their lifestyle in a healthy way and to maintain this new healthy lifestyle. During a CLI, patients are supported by healthcare professionals to create a healthy lifestyle and to get tools to adhere to this healthy lifestyle. A CLI consists of advice and counseling to improve health related-behaviors as physical activity and eating habits. Lack of physical activity is associated with a less favorable progress in DM2 disease course and an increase in all-cause mortality rates [4]. Moreover, patients already diagnosed with DM2 or CVD have a high prevalence of physical inactivity [5]. Hence, lifestyle interventions including attention for physical activity are being implemented to prevent DM2 in high-risk patients and favorably influence the course of disease in DM2 patients. These interventions seem to be at least as effective as pharmacological interventions and reduced the risk of developing diabetes in people with impaired glucose tolerance by about 50% [1]. A CLI aims to improve health-related behavior such as physical activity and diet. By adapting healthy lifestyle habits, complications or worsening of DM2 and/or CVD might be prevented, postponed or even reversed [4, 6]. Effective CLIs will result in increased quality of life for patients and lower medical costs. However, successful implementation of these interventions poses a challenge, since participation rates tend to be low [7, 8]. Reasons for low participation rates might be lack of time, costs of participating or transport issues [9]. Searching for ways to improve participation rates and adherence to prescribed lifestyle interventions, health promoting financial incentives (HPFIs) are implemented as addition to the CLI.

HPFIs are cash or cash-like rewards or fines provided contingent on (non-)performance of healthy behaviors [10]. Besides the two main categories of positive (e.g. reward or discount) or negative (e.g. a fine or higher out of pocket costs) financial incentives, there is great variation in characteristics of an HPFI. Saving campaigns or deposit contracts are also a form of a HPFI. Saving campaigns in general, like collecting loyalty points for free products, are popular in the Netherlands. A deposit contract means that participants of a CLI pay a certain amount of money to participate, and by meeting prerequisites that are determined at the start of the CLI they can get a part of the amount of the whole amount of the deposit back.

The effectiveness of HPFIs added to a CLI on for example participation rates or program adherence is not proven yet. Only a few studies had a good study design and besides some short-term effect, no long-term effects were found [11]. HPFIs are not implemented frequently in the primary care setting in the Netherlands, but there is increasing interest in implementation of HPFIs. For a successful implementation process, it is necessary to have more elaborate knowledge on what the opinions of the stakeholders are. This descriptive qualitative study shows barriers and facilitators in the implementation of a CLI in care groups for patients with DM2 or CVD, as perceived by the stakeholders, with special attention for the supposed influence of adding a HPFI to the CLI on the implementation process [12].

## Methods

### Implementation process CLI and HPFI

Originally, our study aimed to investigate the (cost) effectiveness of adding a HPFI to a CLI.

However, despite great effort within the participating care group to create support for the CLI, and extensive research beforehand on the preferences of the target population regarding optimal characteristics of the HPFI [13], the implementation process of this specific CLI hampered and the number of patients willing and able to participate was too low to start the CLI. As a consequence, the effect evaluation and the cost effectiveness study of that particular CLI was cancelled. Instead we executed a more elaborate and broader process evaluation of CLIs in general in order to learn more on the implementation process of a CLI and the feasibility of implementing a HPFI.

### Design and procedures

A qualitative research design with semi-structured interviews was used to investigate the opinions of professionals involved in six Dutch care groups offering a CLI. This selection of care groups consisted of the particular care group that planned to implement the CLI and a HPFI and did not succeed and five other care groups. General practitioners, practice nurses, representatives of management of the care groups, as well as community health services policy staff related to these care groups were interviewed about barriers and facilitators for implementation of CLIs in primary care for patients with DM2 and/or CVD and about the possibility of complementing these CLIs with a HPFI. The research proposal was reviewed and approved by the Ethical Review Board of the Tilburg University before executing the study.

### Respondents

Care groups eligible for this study had to offer a CLI to patients with DM2 and/or CVD. This intervention had to include both a diet and physical activity component. One researcher (StS) viewed the websites of 94 care groups in the Netherlands and identified twelve care groups that fulfilled these criteria. These care groups differed in size and region. The smallest care group included 11 general practitioners, whereas the largest care group included more than 200 general practitioners. In general, these 12 care groups were mostly located in the southern and western parts of the Netherlands. In total, six care groups were included in this study. Besides the care group that planned to implement the CLI and HPFI, a purposive sample of five care groups was taken out of the twelve other care groups identified to be eligible and proven to be willing to participate. This resulted in six care groups in total that varied in size.

Potential respondents were invited to participate in the study via email. If no response by email was received, they were contacted by telephone. In total 24 interviews (at least 3 persons per care group) were conducted with care group managers, general practitioners, practice nurses, community health services policy staff related to the care groups and one manager of a health care insurer. Table 1 shows the respondents for each of the six care groups.

**Table 1:** Respondents per care group

Care group	GP	Practice nurse	Representative management of care group	Community health services policy staff^a^	Other
1^b^	X	X^c^	X	X	1 dietician, 1 physiotherapist, 1 health care insurer
2^d^	X	X	X		
3	X	X	X	X	
4	X		X	X	
5	X	X	X	X	
6		X	X	X	

^a^1 community health service had 2 care groups in its region. In total 4 interviews with community health services were performed

^b^ Care group of the original study region

^c^ 3 practice nurses were interviewed from the care group of the original study region

^d^ The GP and practice nurse from this care group were interviewed together

### Research questions

The interview guide (Additional file 1) was set up to provide information with regard to following research questions:

- What are preferred characteristics of a CLI and factors expected to influence implementation of a CLI according to the respondents?
- What are preconditions for successful implementation of a CLI?
- What are preferred characteristics and the expected effect of HPFIs when added to a CLI according to the respondents?
- What is the attitude of the respondents towards HPFIs in relation to a CLI?

### Data collection

Semi-structured interviews were conducted mostly face-to-face and some by telephone. Data collection took place between March and October 2017. An interview guide was used to discuss the barriers, facilitators and experiences with implementation of a CLI, the role HPFIs can play, opinions with regard to the content and effectiveness of HPFIs and the expectations for the future with regard to CLIs and HPFIs for patients with DM2 and CVD in primary care.

Before the start of the interview, respondents signed a written informed consent, agreeing to participate in the study and to the audio recording of the interview. The interviews were held in private, so the respondent could speak freely. The interviews lasted between 30 to 60 min, were transcribed verbatim and were rendered anonymous so that they could not be traced to the respondents. The two interviews were performed by both CM and StS to determine how the design of the interview guide worked out in practice. We did not make adjustments to the interview guide. Researchers StS and TdV performed the following interviews individually. Both interviewers did not have work-related contact with the interviewees and were independent. In this way, we have done our best to ensure respondents would not give social desirable answers. After 24 interviews, no new results were identified and therefore we believe we reached data saturation and stopped the inclusion of care groups for the study.

### Data analysis

The interviews were analyzed using a thematic approach with support of the software program MAXQDA 2018. The focus of the thematic content analysis is coding and analyzing the interviews with regard to themes [14, 15]. First, a more inductive approach was chosen and pieces of text were marked and received summarizing terms like, “long-term vision” or “opinion with regard to financial incentives” [15]. This process of open coding resulted in a list of codes. Secondly, the codes from phase 1 were ordered, deleted or merged with other codes by using axial coding. Moreover, codes were clustered and a distinction was made between main and sub codes, which resulted in a code tree with main codes like, “barriers for implementation” and sub codes like “lack of time”, “” and “funding”. Thirdly, the categories were structured and the most important categories were determined by using selective coding (Appendix 1). Three researchers (StS, CM, WWV) worked independently to analyze data and formed pairs to gain consensus and to guarantee the credibility. If no consensus was achieved, a third researcher (WWV and LvdG) was consulted to reach a final decision.

## Results

First, the preferred characteristics of a CLI, factors expected to influence implementation of a CLI and preconditions for successful implementation of a CLI are presented. Second, insights are given with respect to preferred characteristics of HPFIs, expected effects of HPFIs when added to a CLI and the attitude of respondents towards HPFIs.

### Combined lifestyle interventions (CLIs)

#### Preferred characteristics of CLI

Easy accessibility of the intervention site, in other words being close to home and the content of the CLI is appropriate for everyone, was mentioned by most respondents as a facilitating factor for participation in a CLI, because most patients have little or no intrinsic motivation to go exercising. In rural areas, this would mean ‘within the same village’ and in urban areas ‘within the same neighborhood’. The intervention called ‘Biowalking’ was considered easily accessible, because it has a very low-threshold, just walking in nature with a group of participants. Many respondents had an opinion with regard to group interventions and the social aspect, but these opinions varied and no clear preference for a group or individual intervention was found. Social interaction and connection between group members was suggested as a factor that would foster the adherence of the intervention itself. A group is a binding factor, because it is pleasant, patients support each other and give advice to each other. Group pressure and positive experiences of other patients can be a motivation for participants. At the same time, some participants mentioned that the group setting could also be a barrier for participating in a CLI. Participating in a group intervention can be scary, because new participants are unfamiliar with the rest of the group. A more practical difficulty that was mentioned is that not all participants can be expected to be available at the same moment for the sessions.

Quote 1:

*“We see that the group process and pressure motivates people to exercise, but also to get in touch with others and share information about diabetes or other conditions.”*

A facilitating factor mentioned in the more medical context was the presence of health care professionals during sessions of the CLI. For example, respondents anticipated that DM2 patients might find it a comforting idea that if they get hypoglycemia during physical activity, a health care professional is present to give medical care or advice. Respondents also mentioned that the CLI has to fit to the needs of the target group. For example patients with low health literacy or speaking a foreign language as their native language should be supervised by someone who talks slow and simple language so the patients are able to understand the information given to them. Moreover, the content of the CLI has to fit to the perspective of the patient on what he or she believes is important to improve with regard to their health status and/or health behavior.

In general, respondents were concerned about patients not being able to continue with behavioral change achieved within a CLI after the intervention was finished. An important facilitator in this respect was creating a good transition to regular sports clubs and facilities; i.e. outside of the health care sector.

Many respondents had an opinion on out of pocket costs for the patients, but the opinions of were not univocal. Part of them mentioned out of pocket costs for the patients as a facilitator for participation because it creates motivation for attending sessions and finishing the program. In addition, they expected patients to make a more well-considered decision whether or not to participate in a CLI. On the other hand, a similar number of respondents stated that out of pocket costs might be a barrier for participation, especially for patients with a low budget.

Quote 2:

*“What I actually think, out-of-pocket costs can also stimulate. If you choose to do it and you pay for it, you also have more inclination to go for it. If it is all non-committal, you tend to be less concerned with it, that is the way in which people think. I do not think everything just has to be for free.”*

#### Factors expected to influence implementation of CLI

A facilitating factor that respondents marked as important was that health care professionals know which CLIs are available to offer their patients. Such knowledge, they indicated, is not always present. Another related facilitating factor mentioned was that referral to a CLI is easier if associated health care professionals know each other and already cooperate in a broader sense. Moreover, word of mouth promotion by the participants themselves could also facilitate implementation of a CLI. Respondents pointed out that successful recruitment of participants for the CLI is important. Factors that were mentioned to possibly facilitate recruitment were a recruitment strategy adjusted to the goals of the target group, and personal contact between health care professional and patients. About three quarters of the respondents mentioned that enthusiasm and willingness among health care professionals is very important. Enthusiastic health care professionals tend to propagate successes of the CLI and they tend to take a more active role in identifying eligible patients and offering the CLI to them. Willingness to invest in implementing a CLI and invest time to convince patients to participate in a CLI is important for successful implementation. In addition, timing constituted an important facilitating factor according to the respondents. For example, short after diagnosis of DM2, patients are more prone to participate in a CLI.

Quote 3:

*“I have already noticed in this course, that you are very dependent on the practice nurse and the general practitioner who is in agreement with the patients. And if the practice nurse or general practitioner does not believe in the program or only sees obstacles, they are less motivated to motivate the patients. I saw the effect.”*

Over half of the respondents mentioned lack of (long-term) funding as an important barrier for implementation. In general, respondents felt that health care insurers are not very keen on financing a CLI or another form of prevention programs. If funding was available most of the time it was temporarily. In this case they experienced that when funding stopped, the CLI also stopped. Other factors mentioned are lack of ownership and lack of time. According to almost all respondents, a barrier for implementation for a CLI is that health care professionals don not always tend to consider themselves responsible for offering a CLI and they state a lack of time to present it to their patients. At the same time, a few respondents mentioned that practice nurses are more willing than GPs to implement a CLI in their practice even though lack of time is also an important barrier for them. Another barrier that was mentioned was that CLIs often are not tailored to hard-to-reach groups, such as (female) immigrants who do not speak the Dutch language or patients who only visit the GP practice once a year. About half of the respondents also pointed out that in some cases health care professionals tend to decide beforehand which patients they believe do not want to participate in a CLI for multiple reasons, resulting in non-referral. They all agreed this is not desirable. Low or no inflow of participants was also mentioned as a barrier for implementation. Patient-related factors that were identified were lack of motivation to put effort in their own health and lack of time. Two respondents suggested a quite radical change of the integrated care program to support the implementation of a CLI. Instead of having a care program aiming at managing one particular disease such as DM2 or CVD, which is the current situation in the Netherlands, they suggested a mandatory prevention program initiated by health care professionals aiming to prevent chronic disease from a healthy lifestyle perspective. This prevention program should not have a single disease focus like the programs that are nowadays applicable in primary care in the Netherlands.

#### Preconditions for successful implementation of CLI

When explicitly asked, the health care professionals suggested several essential preconditions for successful implementation of a CLI. The following preconditions are sorted by mentioned most to mentioned least:

- Funding which has to be arranged and clear beforehand.
- The CLI has to fit the needs of the target group. This might demand a custom-made program per participant.
- Incorporating proven effective elements in a well-considered plan for the content of a CLI.
- A good infrastructure and communication between all stakeholders.
- Motivated and enthusiastic health care professionals, as well as constructive collaboration between all different health care professionals.
- A tailored recruitment strategy resulting in a continuous inflow of eligible participants.

### Health promoting financial incentives (HPFIs)

#### Preferred characteristics of HPFIs

Overall, positive HPFIs were preferred over negative HPFIs. Respondents expected negative HPFIs (e.g. pay a fine or other extra costs) to raise aversion with the patients resulting in them choosing not to participate in the CLI at all. Especially for patients with a low budget they expected this fine or extra costs to be a large threshold.

Quote 4:

*“That punishment does not seem the solution to me. I mean, if you tell me: ‘if you do not come, you have to pay a fine’. Then I say: ‘well, I will not participate at all”.*

Respondents suggested several forms of positive HPFIs, such as a discount on their health insurance fee, participating in a CLI without costs, saving campaigns, deposit contracts and discounts on or freely available fruit and vegetables, or free sports materials. Some of the respondents suggested that the positive HPFI should not be given at the end of a program, but divided over times in small steps. Saving campaigns (e.g. loyalty points for free products) were particularly mentioned, because this type of HPFI is already familiar to most people and they expected long-term effects when a HPFI would link with systems and processes already effectively implemented in the daily lives of people. Deposit contracts were also among the frequently mentioned preferred characteristics of HPFIs. In this case, patients pay to participate in a CLI and could regain (a part of) the amount paid for example by attending all appointments.

#### Expected effects of HPFIs

About half of the respondents mentioned that adding a positive HPFI to a CLI could potentially break down barriers for patients to enroll in a CLI and even positively influence the amount of participants finishing the program. Majority of the respondents touched on the discussion whether an extrinsic motivator such as a HPFI could be the key to building intrinsic motivation for behavioral change. By breaking down the barriers to participate, enrolled patients might experience the effects of more exercise and healthy nutrition on their health. As a consequence of feeling better, they may continue the program or their adapted behavior. However, in general the respondents did not expect HPFIs to generate long-term effects. They expected most patients to revert to their old habits after completing a CLI, despite the HPFI. They suggested a continuous stimulant in the form of a HPFI or regular checks by a health care professional to prevent relapse. Specifically for patients with a low budget, they thought it would be helpful if the HPFI could take the form of a fully reimbursed program.

Quotes 5 and 6:

*“Well, I think motivation can be bought.”*

*“No, I do not believe in financial incentives. I think that the only effective incentive is a social one.”*

#### Attitude towards HPFIs

Overall, the attitude of the respondents with regard to adding a HPFI to a CLI diverged. Most respondents preferred participants in a CLI who have intrinsic motivation to participate, instead of participants who only participate in the CLI because they get a reward. There were also some respondents with a more positive attitude towards adding a HPFI to a CLI. They mentioned that they believed that patients would appreciate the reward for their efforts.

With regard to future implementation of HPFIs, respondents had different opinions. Part of them was convinced that it is more important to have an easy accessible CLI than to extrinsically motivate patients with a HPFI. Feasible forms of HPFIs mentioned were discount on the health care insurance or it could be that patients were exempted for paying out of pocket costs to participate in a CLI. In their opinion, collaboration with employers, industry and stores might help to fund HPFIs on a broader scale.

## Discussion

In this study, we have evaluated perceived barriers and facilitators associated with the process of implementation of a CLI in primary care for patients with DM2 or CVD, the preferred characteristics of both a CLI and HPFI, with special attention for the influence of adding a HPFI to the CLI on the implementation process. To this aim, we interviewed health care professionals and other stakeholders from six care groups, such as a care group manager and a manager of a health care insurance company.

Preferred characteristics of the CLI, according to our respondents were easy accessibility, the presence of health care professionals during for example exercise sessions, content of the CLI fitted to what the patient believes is important to improve with regard to their health status and health behavior. Opinions were not univocal for out of pocket costs and a structure of the CLI with group consults. Factors promoting the implementation of a CLI according to the respondents were often related to attitude and behavior of health care professionals. Perceived facilitating factors mentioned were enthusiastic health care professionals, knowledge with regard to the CLI, and health care professionals involved in the CLI already knowing each other and cooperating in a broader sense. Preconditions for a successful implementation of a CLI mentioned were structural funding, good infrastructure and communication between stakeholders, the CLI being tailored to the needs of the target group, motivated health care professionals and a tailored recruitment strategy.

As to the HPFIs, respondents preferred positive HPFIs to negative HPFIs and generally agreed that adding a HPFI to a CLI could potentially break down barriers for patients to enroll in a CLI. A focus group study including the general public also showed that positive HPFIs are preferred [16]. They also expected it might have a positive influence on the degree of actual participation in the CLI and possibly even finishing the program. However, the respondents also questioned if an extrinsic motivator could be the key to achieve long-term behavioral change. Long-term effects of HPFIs were not expected.

A lack of time of the health care professionals was mentioned as a perceived barrier to offer CLIs to the patients. This perceived barrier was already mentioned in other studies and implies that a change is needed in the workload of the GP and practice nurse [17–19]. The study of the “Beweegkuur” (i.e. exercise on prescription) showed that out of pocket costs were a barrier for the patients to participate [17]. The respondents in our study shared this opinion, but it was also mentioned that out of pocket costs could help to make a more well considered decision to participate in a CLI. This contradiction was also found in the study of Geense et al. [19]. In our study, some respondents mentioned that prevention is mainly seen as a task for the practice nurse. Practice nurses have more time to explain the CLI to the patient, and in the Dutch system DM2 and CVD patients have most of their checkups at the practice nurse. However, the qualitative study of Helmink et al. found though that it could be useful to let the GP ask the patient to participate in the CLI [17] because of the doctors’ natural authority. The study of Geense et al. showed, comparable to our results, that the availability of a practice nurse is a facilitating factor for implementing lifestyle programs and that the attitude of GPs towards lifestyle interventions differ [19].

Patient engagement and paternalism in health care is addressed last decades more and more [20–22]. Shared decision making and patient centeredness is preferred over paternalism of the health care professional. Our study show that paternalism is still present in primary health care, because respondents mention that health care professionals tend to decide on beforehand which patients they believe would or would not participate in a CLI, without asking the patient. This is not desirable as patients are now excluded from participation while they might have motivation to participate. On beforehand of implementing a CLI, more attention should be paid to the process of shared decision making and patient engagement. The study of Elwyn et al. show that the degree of patient involvement will depend on the skills and attitude of the health care professional [23]. Possibly more education is necessary to develop the necessary skills and attitude of the health care professionals before implementing a CLI.

The number of studies that evaluated projects in which a HPFI was implemented to improve health behavior of patients is growing and results with regard to effectiveness is not univocal [24–26]. Despite the scientific interest in this instrument, most respondents in our study were critical with regard to HPFIs. The overall opinion was that an incentive would only be effective in the short term. Their opinion is in line with most results in the studies mentioned and the argumentation for savings systems, which could influence the participant in the long term, seems plausible [26, 27].

The implementation of an innovation, especially a preventive intervention such as a CLI, in health care is complex. As to the implementation process, the model of Fleuren et al. states that different determinants could influence the implementation process of innovations in health care [28]. Among these, characteristics of the person adopting the innovations and characteristics of the socio-political context, are important to mention. With regard of the characteristics of the person adopting the innovation, in this study the barrier was brought up that health care professionals were not always motivated to implement a CLI or did not see prevention as their task. This also relates to aspects of reimbursement and financing of preventive tasks within our health care system. With respect to the implementation of a CLI placed in the socio-political context, financing of the CLI is a perceived barrier. To be able to overcome all these difficulties, a long-term view and motivation of all stakeholders is important. It is relevant to identify the difficulties that can be expected and that a long-term view is necessary, so expectations of the stakeholders with regard to the implementation process are realistic.

Overall, many facilitators and barriers for successful implementation were identified in this paper. To design a CLI and successfully implement the CLI is not an easy task. Michie et al. have designed a framework called the Behavioural Change Wheel, which is based on the COM-B model [29]. This framework can help to design a CLI that can successfully achieve behavior change. The COM-B model shows the complexity of behavior change and the many factors that play a role. The components capability, motivation, and opportunity interact to create behavior and this behavior then influence the components. The results of our study on facilitators and barriers for implementation of a CLI is diverse, but seem consistent to the complex model of Michie et al. It is advisable to keep in mind the Behavioural Change Wheel when designing a CLI and the implementation process, to maximize the chance of success.

We have interviewed people with different perspectives to get the overall picture and to be able to identify similarities and differences in opinions of different stakeholders. Besides the health care professionals, we have also interviewed management representatives per care group. The opinion of the management of the care group is important, because they can play a role in the implementation process of a lifestyle intervention by helping to set preconditions and eliminate barriers for the implementation of a CLI. Managers often have a clear vison on the development of the care their care group should provide to their patients in the coming years.

Overall, no noticeable differences were found between the respondents of the care group in which originally the CLI with the HPFI would be implemented and the other care groups, which only offered a CLI. Therefore, all results were collectively discussed, also with regard to the addition of a HPFI.

Since this study focused on the implementation process of a CLI by the primary care group we have not included the patients in this study who have participated in a CLI or who were eligible to participate in a CLI. Further research should also have attention for this group. Due to the complexity of the subject, a focus group study might be more appropriate for this target group. Further research on the attitude of the end users towards HPFIs is necessary to generate more detailed knowledge on the settings in which HPFIs are appropriate to use and in which it might be counterproductive. Moreover, implementation of a CLI will be only successful if the characteristics of the CLI and the eventual HPFI are adjusted to the preferences of the end user.

## Conclusion

Overall, we have identified important perceived barriers and facilitators for a successful implementation of a CLI in a primary care setting. Essential preconditions such as structural funding, tailored recruitment strategy, a good infrastructure and communication between all stakeholders, and a good fit of the CLI to the needs of the target group are important for a successful implementation of a CLI. Another relevant factor is that it seems that a shift in attitude of the health care providers is necessary with regard to prevention in general and CLIs in specific. It should be considered as basic care instead of an additional task, which is voluntary to execute if the health care professional has time. For successful implementing CLIs in the future, a more positive attitude of all stakeholders towards CLIs is essential and it should not be without obligations for the health care professionals to offer CLIs to their patients.

A HPFI is an instrument that is not used commonly yet and the health care professionals in our study were somewhat skeptical about the effectiveness. In order to motivate health care professionals who are involved in the execution of a CLI to have a positive attitude towards an HPFI it might be helpful get more insight how health care professionals and the participants of the CLI can be more directly involved in the process of designing a HPFI.

### Supplementary information

Additional file 1. Interview guides.

## Data Availability

In the agreement with the consortium it was stated that data used and/or analysed during the current study are available from the corresponding author on reasonable request. The data will nog be shared on an open data source, because the transcripts were written in Dutch, which makes them available only for Dutch speaking researchers. Moreover, the transcripts do contain information that makes it possible to reduce the data to specific persons, which is not desirable.

## Appendix

**Table 2:** Coding tree

Lifestyle intervention	Attitude interviewee	Future financial incentive added to lifestyle intervention
		Future lifestyle interventions
		Division of tasks
		Out of pocket costs
	Characteristics	Long term
		Awareness
		Own initiative after completing CLI
		Building intrinsic motivation
	Facilitators implementation	Willingness health care insurer
		Attitude patient
		Publicity
		Funding
		Leader
		Enthusiastic health care professionals
		Willingness health care providers
		Mandatory participation
		Long-term vision
		Health care professionals interested in CLI
		Vision of health care providers on their tasks
		Chain cooperation
		Early referral
		Recruitment strategy
		Allocation of tasks
	Barriers implementation	No inflow patients
		Attitude patient
		Complexity
		Funding
		Lack of time
		Willingness health care providers
		Contact with general practitioners
		Willingness of municipalities
		Publicity
		Short-term vision
		Hard to reach target group
		Vision health care professionals
	Preconditions implementation	Enthusiasm
		Fitting the needs of the target group
		Funding
		Well considered content
		Long-term vision
		Recruitment strategy
Willingness to participate in lifestyle intervention	Facilitating characteristics participants	Enthusiasm
		Motivation
		Be open to new ideas
		Having insight
	Hampering characteristics participants	Loneliness
		Having no insight
		Laziness
		Behavioral change is difficult
		Personal problems
		No experience/no knowledge
		Low health literacy/speaking a foreign language
	Facilitating factors lifestyle intervention	Fits needs of the target group
		Continuity
		Social aspect
		Social support
		Set targets
		Out of pocket costs
		Close to home
		Easy accessible
		Available for everyone
		Group/individual program
		Low threshold
		Presence health care professional
		Presence social map
		Presence care sport connectors
		Recruitment strategy
	Hampering characteristics lifestyle intervention	Out of pocket costs
		Not close to home
		No continuity
		Group/individual program
Financial incentive	Characteristics	Provider
		Attitude health care professionals
		Positive incentive
		Negative incentive
		Gamification
	Effect	Motivation
		Lowering threshold
		Giving insight
		Long-term
		Short-term

## Abbreviations

CLI: combined lifestyle intervention
CVD: cardiovascular disease
DM2: diabetes mellitus type 2
GP: general practitioner
HPFI: health promoting financial incentive
