# Supplementary material for: Perceived barriers and facilitators of the implementation of a combined lifestyle intervention with a financial incentive for chronically ill patients
Source: BMC Fam Pract. 2019 Oct 18;20:137. doi: 10.1186/s12875-019-1025-5 (PMC6798404; doi:10.1186/s12875-019-1025-5)
Supplement: Supplementary file 1 — Interview guides. [file 12875_2019_1025_MOESM1_ESM.docx]

**Appendix 1 Interview guides**

Interview guide for health care providers of the care group that participated in the original research**.**

1. What was your role in the implementation process of the combined lifestyle intervention (CLI)?
2. In your opinion, were there barriers or facilitators for the implementation of the CLI? If yes, which one?
3. What was your attitude towards implementing a CLI in the region of the care group?
4. How did you experience the implementation of the CLI? What factors contributed that you experienced it this way?
5. In your opinion, what was the attitude of the care providers concerned to the GLI?
6. Were there differences between the different groups of care providers with regard to their attitude towards the GLI? And if so, which ones?
7. How was the attitude of the health care insurers towards the implementation of a CLI according to you?
8. What do you think are preconditions for a successful implementation of the CLI?
9. To what extent has adding a financial incentive to the CLI, influenced the implementation process of the CLI according to you?
10. What was your attitude towards the financial incentive that was added to the CLI?
11. What do you consider the attitude of the (other) healthcare providers to the financial incentive?
12. The financial incentive intended to motivate participants to participate in and to finish the CLI. Do you think that this specific financial incentive could indeed achieve this?
13. What should be the design of a financial incentive to be effective in your opinion?
14. The intake of participants in the CLI did not go as expected. What do you think are reasons that participation rates fell short of expectations?
15. The CLI was implemented in your care group, but did not succeed. What lessons can be learned from the implementation process of the?
16. What lessons can be learned from the implementation process of the financial incentive?
17. The intake of participants in the CLI did not go as expected. In the future, what do you expect from the supply and demand for lifestyle interventions for physical activity and healthy eating?
18. What do you expect in the future of providing financial incentives for lifestyle interventions on physical activity and healthy eating?
19. Do you expect financial incentives to be used more often in lifestyle interventions on physical activity and healthy eating?
20. What do you expect is the effect to implement lifestyle interventions around physical activity and healthy eating?

Interview guide for health care providers of care groups not participating in the original research.

1. What is your position in your organization?
2. Are there currently lifestyle interventions implemented in your care group aiming to improve physical activity levels of the participant and healthy eating. What is the design of these lifestyle interventions?
3. What factors have promoted the implementation of lifestyle interventions for physical activity and healthy eating in your region?
4. What factors have hampered the implementation of lifestyle interventions for physical activity and healthy eating in your region?
5. In your opinion, what are the factors that hamper or promote the implementation of lifestyle interventions for physical activity and healthy eating?
6. How do you feel about the implementation of lifestyle interventions in your region?
7. What is the attitude of other care providers regarding the implementation of lifestyle interventions on physical activity and healthy eating?
8. Are there differences between the different groups of care providers with regard to their attitude towards lifestyle interventions concerning physical activity and healthy eating? And if so, which ones?
9. What is, according to you, the attitude of health insurers to the implementation of lifestyle interventions regarding physical activity and healthy eating? What do you think of this attitude?
10. What do you think are preconditions for a successful implementation of lifestyle interventions on physical activity and healthy eating?

*Before asking following questions, the interviewers give an explanation about the concept ‘financial incentive’*

1. To what extent do you think that adding a financial incentive for the participants of the lifestyle intervention influences the implementation of lifestyle interventions on physical activity and healthy eating?
2. To what extent do you think that adding a financial incentive to the participants can influence the participation rates or the effectiveness of a lifestyle intervention on physical activity and healthy eating?
3. What is your attitude towards adding a financial incentive to a lifestyle intervention on physical activity and healthy eating?
4. What is, according to you, the attitude of (other) caregivers towards the use of a financial incentive for the participants of the lifestyle intervention?
5. To what extent do you think that the use of a financial incentive can be effective?
6. What should be characteristics of a financial incentive to be effective in your opinion?
7. In the future, what do you expect from the supply and demand for lifestyle interventions for physical activity and healthy eating?
8. What do you expect in the future of providing financial incentives for lifestyle interventions on physical activity and healthy eating?
9. Do you expect financial incentives to be used more often in lifestyle interventions on physical activity and healthy eating?
10. What do you expect is the effect to implement lifestyle interventions around physical activity and healthy eating?

Interview guide for community health services policy staff.

1. What is the role of the community health service regarding the implementation of lifestyle interventions on physical activity and healthy eating?
2. What is your position in your organization?
3. Are there currently lifestyle interventions implemented in your region aiming to improve physical activity levels of the participant and healthy eating. What is the design of these lifestyle interventions?
4. What factors have promoted the implementation of lifestyle interventions for physical activity and healthy eating in your region?
5. What factors have hampered the implementation of lifestyle interventions for physical activity and healthy eating in your region?
6. Could the community health service have had a role in order to remove this barrier? If so what, if not why not?
7. In your opinion, what are the factors that hamper or promote the implementation of lifestyle interventions for physical activity and healthy eating?
8. How do you feel about the implementation of lifestyle interventions in your region?
9. What is the attitude of other care providers regarding the implementation of lifestyle interventions on physical activity and healthy eating?
10. Are there differences between the different groups of care providers with regard to their attitude towards lifestyle interventions concerning physical activity and healthy eating? And if so, which ones?
11. What is, according to you, the attitude of health insurers to the implementation of lifestyle interventions regarding physical activity and healthy eating? What do you think of this attitude?
12. What do you think are preconditions for a successful implementation of lifestyle interventions on physical activity and healthy eating?
13. To what extent do you think that adding a financial incentive for the participants of the lifestyle intervention influences the implementation of lifestyle interventions on physical activity and healthy eating?
14. To what extent do you think that adding a financial incentive to the participants can influence the participation rates or the effectiveness of a lifestyle intervention on physical activity and healthy eating?
15. What is your attitude towards adding a financial incentive to a lifestyle intervention on physical activity and healthy eating?
16. What is, according to you, the attitude of (other) caregivers towards the use of a financial incentive for the participants of the lifestyle intervention?
17. To what extent do you think that the use of a financial incentive can be effective?
18. What should be characteristics of a financial incentive to be effective in your opinion?
19. In the future, what do you expect from the supply and demand for lifestyle interventions on physical activity and healthy eating?
20. What do you expect in the future that the role of the community health service can be in the implementation process of lifestyle interventions on physical activity and healthy eating?
21. What do you expect in the future of providing financial incentives for lifestyle interventions on physical activity and healthy eating?
22. Do you expect financial incentives to be used more often in lifestyle interventions on physical activity and healthy eating? Followed by asking why yes or no
23. What do you expect is the effect to implement lifestyle interventions around physical activity and healthy eating?
